# The Effect of Periodic Assessments and Verbal Feedback on Physical Function and Adherence in Healthy Adults Aged ≥65: A Pilot Randomized Controlled Trial

**DOI:** 10.3390/jfmk11030248

**Published:** 2026-06-25

**Authors:** Danai Paleta, George Gioftsos, Stefanos Karanasios, Panagiotis Paletas, Vasiliki Sakellari

**Affiliations:** 1 MSc Program “New Methods in Physiotherapy”, Department of Physiotherapy, School of Health and Care Sciences, University of West Attica, 12243 Athens, Greece; gioftsos@uniwa.gr (G.G.); skaranasios@uniwa.gr (S.K.); 2 B’ KAPI Renti, Municipality of Nikaia–Agios Ioannis Rentis, 18233 Athens, Greece

**Keywords:** physiotherapy, kinesiology, functional performance, balance, gait, motor control, exercise adherence, older adults, randomized controlled trial, rehabilitation

## Abstract

**Background and Objectives**: Low participation rates in exercise programs among older adults highlight the need for theory-driven, biopsychosocial interventions that enhance adherence, self-efficacy, and functional outcomes. Grounded in principles of motor learning and behavioral reinforcement within physiotherapy practice, this study aimed to examine the effect of periodic assessments combined with verbal feedback on functional and psychological outcomes in community-dwelling older adults. **Methods**: A pilot RCT was conducted involving 54 individuals aged ≥65 years (53 women and 1 man), recruited from senior community centers. Participants were randomly allocated to an intervention group (periodic assessment and verbal feedback; *n* = 27) or a control group (*n* = 27). Both groups participated in an identical 12-week structured exercise program, delivered twice weekly, focusing on balance, gait, and lower-limb functional training. An intention-to-treat approach was applied. Data were analyzed using Linear Mixed Models, with statistical significance set at *p* < 0.05. **Results**: Significant group × time interactions were observed in favor of the intervention group for key kinesiology-related functional outcomes, including the Short Physical Performance Battery (SPPB; *p* < 0.001), Timed Up and Go test (TUG; *p* = 0.011), and Activities-specific Balance Confidence Scale (ABC; *p* < 0.001). No statistically significant differences were identified between groups for the Behavioral Regulation in Exercise Questionnaire–2 (BREQ-2; *p* = 0.164) and the Self-Efficacy for Exercise Scale (ESE; *p* = 0.108), indicating that the primary psychological outcome (ESE) was not confirmed. However, both ESE and BREQ-2 demonstrated significant baseline differences favoring the intervention group, and, therefore, these findings should be interpreted with caution despite statistical adjustment. **Conclusions**: Periodic assessments followed by verbal feedback appear to selectively improve the functional effectiveness of structured exercise programs in older women, particularly physical performance, functional mobility, and balance confidence, with no significant differential effect on the primary psychological outcome (ESE; group × time interaction: *p* = 0.108). These findings support assessment-informed and feedback-driven physiotherapy strategies as a promising adjunct to exercise programs in older adults, with potential implications for optimizing functional outcomes within applied kinesiology and rehabilitation contexts.

## 1. Introduction

### 1.1. Background

The global population is aging rapidly, with older adults aged ≥65 years representing an increasingly large proportion of society [1], thereby posing significant challenges for healthcare systems and public health policy. In this context, healthy aging has emerged as a primary public health policy objective, with regular physical activity recognized as a key determinant of its achievement [2,3]. Extensive evidence suggests that systematic physical activity is associated with improved physical fitness, functional capacity, and psychological and cognitive health in older adults, contributing to the maintenance of independence, quality of life, and healthy aging [4]. Exercise-induced improvements in physical function among older adults are thought to be mediated by neuromuscular and sensorimotor adaptations [5,6]. At a mechanistic level, resistance and multicomponent training have been shown to enhance motor unit recruitment, voluntary activation, and neuromuscular efficiency, reflecting adaptations at both peripheral and central levels of the nervous system [6]. Additionally, neuromuscular training appears to improve sensory integration, cortical activation, and muscular responsiveness, contributing to better balance performance [7]. Despite these well-established benefits, a considerable proportion of older individuals fail to meet recommended activity levels [4], resulting in increased risks of falls, fractures, disability, dementia, depression, cognitive decline, and mortality [8].

Long-term adherence to exercise remains a major challenge in geriatric populations. Within biopsychosocial models of health, exercise participation is influenced by the dynamic interaction of behavioral, environmental, and psychosocial factors [9,10,11]. Recent evidence indicates that psychological variables are identified as strong predictors of participation, with self-efficacy emerging as the strongest predictor of exercise initiation and maintenance [12,13]. Within the framework of A. Bandura’s Social Cognitive Theory [10], self-efficacy reflects an individual’s belief in their ability to successfully perform specific behaviors and is shaped by four primary sources: mastery experiences, vicarious experiences, psychological and emotional states, and verbal persuasion [14]. As core constructs of this theory, self-perceived competence and self-efficacy reflect the individual’s confidence in their ability to sustain exercise participation and have been shown to exert stronger positive effects on the maintenance of physical activity, compared with other psychological variables [13,15].

### 1.2. Theoretical Background

Recent evidence supports a paradigm shift from purely biomedical models towards biopsychosocial frameworks, promoting a holistic approach and emphasizing the recognition of disability and functional limitations as dynamic, multifactorial outcomes arising from the interaction of biological deficits, psychological state, and environmental factors [9,12,16]. Within this context, the International Classification of Functioning, Disability and Health [9] provides a comprehensive framework for understanding functional capacity as a dynamic interaction between body functions, activities, participation, and contextual factors [9]. This framework is particularly relevant in older adults and has been successfully applied in both clinical and research settings in geriatric populations [12,17]. Thus, according to the adapted ICF framework for older adults, psychological, social, and environmental factors appear to act either as facilitators or barriers to participation in physical activity and consequently influence functional capacity and disability [16,18,19].

Task-specific training further facilitates motor learning processes in older adults, as repeated practice of functionally relevant tasks promotes the acquisition, refinement, and retention of motor skills. Motor learning is defined as a process through which movements become more efficient and automated with practice, involving both performance improvements and consolidation mechanisms [20]. Importantly, interventions incorporating task-oriented and dual-task paradigms have been shown to improve postural stability and mobility, suggesting that repeated exposure to ecologically valid motor demands may enhance the development of more efficient movement strategies [21]. In parallel, sensorimotor integration appears to be refined through repeated exposure to multimodal sensory inputs, including proprioceptive, vestibular, and visual information. Training programs targeting postural control and proprioception demonstrate significant improvements in proprioceptive accuracy and balance performance in older adults, supporting the role of repeated sensory-motor stimulation in optimizing afferent processing and motor output [22]. At a neurophysiological level, evidence indicates that motor practice, when combined with physical exercise, can enhance motor skill learning in older adults while increasing corticospinal responsiveness [23].

A. Bandura’s Social Cognitive Theory (1997) adds a behavioral dimension to the biopsychosocial model by highlighting perceived self-efficacy as a key determinant in predicting health behaviors and a key mechanism underlying engagement in physical activity [24]. The development of self-efficacy is based on four primary sources that interact to shape an individual’s belief in their ability to achieve specific goals [14]. One of these sources, verbal persuasion, particularly when delivered clearly and credibly by qualified professionals, can reinforce self-belief and support engagement in goal-directed health behaviors [14]. The presence of an instructor providing real-time feedback in group-based physical activity programs has been shown to enhance motivation, adherence, and participant engagement in exercise [11,25]. The enhancement of self-efficacy within exercise programs, through increased confidence in performing exercises and managing perceived barriers, has also been associated with higher levels of physical activity participation and long-term adherence, supporting the practical application of Social Cognitive Theory in the design of effective exercise interventions [26,27,28]. The integration of periodic assessments and structured verbal feedback is hypothesized to potentiate these mechanisms by increasing attentional focus, reinforcing correct movement execution, and promoting error-based learning. This may be particularly relevant for older adults, where external feedback can compensate for age-related declines in intrinsic feedback processing, ultimately enhancing adherence and self-perceived competence.

### 1.3. Aims

The provision of verbal encouragement based on periodic assessments represents a direct application of the verbal persuasion pillar of Bandura’s theory—the delivery of specific, positive, and credible feedback from a qualified health professional—and is proposed to reinforce self-efficacy and, consequently, exercise adherence and functional improvement. Despite the shift from a strictly biomedical model toward a biopsychosocial framework and the growing body of research identifying determinants of exercise participation in older adults, the specific role of periodic assessment combined with verbal feedback during a structured exercise program, grounded in Bandura’s Social Cognitive Theory (1997), remains underexplored in the international literature. Therefore, the present pilot randomized controlled trial was designed to investigate the effects of periodic evaluation and verbal feedback during a 12-week supervised exercise program in healthy community-dwelling older adults ≥65 years, focusing on functional capacity, exercise adherence, and exercise-related self-efficacy, compared with the same program delivered without feedback and reinforcement.

The study aimed to compare two different approaches in this geriatric population regarding their effects on: (a) physical fitness and functional capacity (SPPB); (b) self-efficacy for exercise (ESE); (c) functional mobility (TUG); (d) balance confidence (ABC); (e) exercise motivation (BREQ-2); and (f) exercise adherence.

### 1.4. Research Hypothesis

Null hypothesis (H0): Periodic assessment and verbal feedback will not produce different effects on functional capacity, exercise adherence, and self-perceived competence in healthy adults aged ≥65 years participating in an exercise program, compared to no assessment or feedback.

Alternative hypothesis (H1): Periodic assessment and verbal feedback will produce different effects on functional capacity, exercise adherence, and self-perceived competence in healthy adults aged ≥65 years participating in an exercise program, compared to no assessment or feedback.

## 2. Materials & Methods

### 2.1. Study Protocol and Ethical Approval

This study was a single-blinded, parallel-group, pilot randomized controlled trial. The research protocol received ethical approval from the Ethics and Research Committee of the University of West Attica, Greece (Protocol number: 107620/24-10-2025), and was conducted in accordance with the Declaration of Helsinki [29]. All participants provided written informed consent prior to enrollment. The study followed CONSORT 2010 reporting guidelines. Furthermore, the clinical trial was registered on ClinicalTrials.gov (NCT07437521).

### 2.2. Participants—Eligibility Criteria

The present study was conducted at senior community centers (KAPI) of the Municipality of Nikaia–Agios Ioannis Rentis, Athens, Greece. Recruitment was conducted through oral presentations and written materials distributed across the KAPI units. Participants who expressed interest were eligible if they met the following criteria: (i) community-dwelling adults aged ≥65 years; (ii) of either sex; (iii) actively registered members of community centers (KAPI) of the Municipality of Nikaia–Agios Ioannis Rentis; (iv) living independently; and (v) able to stand, ambulate, and perform activities of daily living without assistance. Exclusion criteria involved: (i) medical advice against participation in exercise; (ii) presence of severe underlying medical conditions; (iii) significant limitations in functional capacity; (iv) inability to complete the Short Physical Performance Battery (SPPB) [30]; (v) hypotension or orthostatic hypotension; (vi) diagnosed psychiatric disorders; (vii) cognitive impairment (Mini-Mental State Examination score < 24/30) [31]; (viii) significant visual or hearing impairments; and (ix) use of medication that could limit safe participation in exercise.

### 2.3. Sample Size

Given the pilot nature of the present randomized controlled trial, no formal a priori power calculation was performed. The primary aim of the study was to assess feasibility, acceptability, and preliminary estimates of intervention effects, in line with established methodological recommendations for pilot trials [32]. The sample size was therefore determined pragmatically, based on feasibility considerations and consistency with previous pilot studies in community-dwelling older adults using comparable outcome measures [33,34,35,36]. As such, the study was not powered to detect small-to-moderate between-group differences, particularly for psychological outcomes such as ESE and BREQ-2. Consequently, non-significant findings for these outcomes should not be interpreted as evidence of the absence of effect or equivalence between groups, but rather as inconclusive results requiring confirmation in adequately powered trials.

### 2.4. Randomization and Blinding

Participants were randomly allocated in a 1:1 ratio to either (i) the intervention group or (ii) the control group, using a computer-generated randomization sequence “http://www.randomization.com (accessed on 20 October 2025)”, performed by an independent, non-clinical researcher, ensuring allocation concealment. Following randomization, participant data were coded using numerical identifiers. Participants were not blinded to their group allocation, whereas the physiotherapist delivering the exercise program was blinded to group assignment. Participants were instructed not to disclose their group allocation, and intermediate assessments were scheduled on different days from the exercise sessions, ensuring that the physiotherapist delivering the exercise intervention was not present at the facility during these assessments. However, full blinding cannot be guaranteed. The assessor physiotherapist who conducted all the assessments -baseline, interim, post- to both groups and provided personalized verbal feedback was not blinded to group allocation. This led to a single-blind design, as complete double-blinding was not feasible given the nature of the intervention.

### 2.5. Intervention and Comparison

#### 2.5.1. Intervention Procedure

Prior to the initiation of the intervention, an informational session was conducted for all participants, regardless of group allocation. During this session, the study procedures and timeline were explained, and participants were instructed to maintain their usual level of daily activities while refraining from participation in any additional structured exercise programs throughout the intervention period. At baseline, a comprehensive assessment was performed, including medical history and demographic data collection (age, sex, height, weight, body mass index, physical activity level, previous exercise participation, smoking status, medication use, and fall history within the past year). Participants completed the Behavioral Regulation in Exercise Questionnaire-2 (BREQ-2), the Activities-specific Balance Confidence (ABC) Scale, and the Self-Efficacy for Exercise (ESE) Scale. Functional performance was assessed using the Short Physical Performance Battery (SPPB) and the Timed Up and Go (TUG) test.

The exercise sessions were delivered twice weekly with a minimum 48 h inter-session interval. Each session lasted approximately 50 min and included a warm-up phase (5–10 min) (in situ walking, upper and lower limb mobility exercises, small-range trunk movements, toe raises, small-range squats); the main exercise program (30 min) (8–10 exercises targeting major muscle groups of the upper and lower limbs, 1–3 sets, 10–15 repetitions, e.g., using elastic resistance bands, dumbbells, or body weight—hip/knee/ankle flexion–extension, shoulder/hip abduction–adduction, wall push-ups, chair stand, step exercises, dynamic and static balance exercises); and a cool-down phase (5–10 min) (upper and lower limb stretching, 30–60 s per stretch). Exercise intensity was set at a moderate level, according to the OMNI-RES perceived exertion scale, with progressive overload applied across the program, in accordance with ACSM guidelines [37]. Sessions were conducted on fixed days and times based on facility availability.

In addition to the common exercise program, participants in the intervention group (*n* = 27) underwent three additional assessments at weeks 3, 6, and 9 [38,39,40], using the Timed Up and Go (TUG) test and the Activities-specific Balance Confidence Scale (ABC), conducted under standardized conditions identical to baseline (same time of day, location, and assessor). Following each assessment, participants received individualized verbal feedback from the same assessor/physiotherapist, including information on performance, progress compared to previous assessments, identification of areas for improvement, and guidance for safe and effective continuation of the exercise program. Feedback was delivered following a consistent structure across participants, in a clear, concise, and encouraging manner, with a duration of approximately 20 min for each participant, by the same assessor/physiotherapist who conducted the assessments. In contrast, participants in the control group (*n* = 27) did not receive interim assessments or individualized verbal feedback regarding their progress throughout the program.

Following the 12-week intervention period, all participants underwent post-intervention assessment, which included repetition of all baseline questionnaires and functional tests. Outcome assessments were conducted under the same standardized conditions as baseline (same time of day, setting, equipment, and assessor) to ensure consistency and reliability of measurements.

#### 2.5.2. Verbal Feedback Procedure

To enhance intervention reproducibility and ensure standardization, the verbal feedback procedure was delivered according to a structured protocol developed a priori by the research team. The feedback content was guided by a predefined framework including four core components: (i) performance feedback (objective results from TUG and ABC assessments), (ii) progress feedback (comparison with previous assessments), (iii) identification of areas for improvement (movement quality, balance strategies, and task execution), and (iv) individualized guidance for safe and effective continuation of the exercise program. All feedback sessions were delivered by the same trained physiotherapist, following a standardized format and sequence to ensure consistency across participants and time points. Although feedback was individualized based on participant performance, its structure and key messages were consistent and aligned with the predefined protocol. To support intervention fidelity, the physiotherapist received prior training on the delivery of the feedback protocol, and a structured checklist was used to ensure that all key components were systematically addressed during each session. Session duration was approximately 20 min per participant.

### 2.6. Outcome Measures

#### 2.6.1. Primary Outcome Measures

Primary outcomes of this study included physical fitness and functional capacity, assessed using the Short Physical Performance Battery (SPPB), and exercise-related self-efficacy, assessed using the Exercise Self-Efficacy Scale (ESE).

1. Physical fitness and functional capacity

The Short Physical Performance Battery (SPPB) is a valid and reliable tool for assessing physical function in community-dwelling older adults [41]. The SPPB evaluates three components: standing balance, gait speed, and lower limb strength (chair stand performance) [42]. Each component is scored from 0 to 4, with a total score ranging from 0 to 12, where higher scores indicate better functional performance. Scores below 10 have been associated with increased fall risk, disability, hospitalization, and mortality [30]. The SPPB was administered at baseline and post-intervention (12 weeks) in both groups.

2. Exercise-related self-efficacy

The Exercise Self-Efficacy Scale (ESE) is a self-reported questionnaire developed to measure confidence in one’s ability to engage in physical activity under various challenging conditions [43]. The scale consists of five items rated on an 11-point Likert scale (from 0 = not confident at all to 10 = very confident) [44]. The Greek version of the scale has demonstrated satisfactory psychometric properties, validity, and reliability for use in Greek adult populations [15]. The ESE was administered at baseline and post-intervention (12 weeks) in both groups.

#### 2.6.2. Secondary Outcome Measures

Secondary outcomes of the present study included exercise adherence, balance confidence (Activities-specific Balance Confidence Scale; ABC), functional mobility (Timed Up and Go test; TUG), and exercise motivation (Behavioral Regulation in Exercise Questionnaire-2; BREQ-2).

1. Functional mobility

The Timed Up and Go (TUG) test is a widely used, valid, and reliable measure of balance, mobility, and fall risk in older adults [45,46]. The test records the time required for a participant to stand up from a chair, walk a distance of 3 m, turn, return to the chair, and sit. Completion times < 10 s are considered indicative of normal mobility in healthy older adults, whereas completion times of >13.5 s have been associated with an increased risk of falls [47]. The TUG test is widely used in both clinical and research settings and has demonstrated high reliability (ICC > 0.95) [48]. The TUG test was administered at baseline and post-intervention for both groups and at weeks 3, 6, and 9 for the intervention group only.

2. Balance confidence

The Activities-specific Balance Confidence (ABC) Scale is a self-reported measure of an individual’s confidence in maintaining balance during the performance of daily functional activities [49]. The scale consists of 16 items describing common activities (e.g., walking on a slippery surface or reaching for an object on the floor), with responses expressed as percentages ranging from 0% (no confidence) to 100% (complete confidence) [49,50,51]. Total scores are interpreted as follows: <50% indicates low functional level, 50–80% moderate level, and >80% high balance confidence [49]. The ABC Scale has been validated for the Greek population and is widely used as a reliable and valid instrument in both healthy older adults and clinical populations with balance impairments [50,52,53,54]. The ABC scale was administered at baseline and post-intervention for both groups, and at weeks 3, 6, and 9 for the intervention group only.

3. Exercise motivation

The Behavioral Regulation in Exercise Questionnaire-2 (BREQ-2) is a widely used instrument for evaluating motivational regulations toward physical activity. The BREQ-2 is an extension of the original BREQ-1 developed by Mullan, Markland, and Ingledew (1997) [55,56] with additional items introduced by Markland and Tobin to assess amotivation. The Greek version of the questionnaire has been translated and validated for use in the Greek population, demonstrating satisfactory psychometric properties, reliability, and validity in adult populations, including older adults [55,56]. The instrument comprises five subscales: amotivation, external regulation, introjected regulation, identified regulation, and intrinsic motivation. Higher scores indicate more self-determined forms of motivation and greater engagement in exercise behavior. The BREQ-2 was administered at baseline and post-intervention (12 weeks) in both groups.

4. Exercise adherence

Exercise adherence was defined at the end of the 12-week intervention as the percentage of completed exercise sessions relative to the total number of prescribed sessions (100%) [57,58]. Adherence was monitored in both groups using standardized attendance logs maintained by the physiotherapist delivering the intervention, ensuring systematic weekly recording of participation. A satisfactory adherence level was defined as attendance of ≥80% of the prescribed sessions, in line with previous studies examining adherence in exercise-based interventions in older adults [57,58].

All assessments were conducted by the same assessor, under identical conditions, at a comparable time of day, in the same setting, and using the same instruments, to ensure measurement consistency and reliability. The assessment timing is shown in Table 1.

### 2.7. Statistical Analysis

Statistical analysis was performed using IBM SPSS Statistics (version 29.0), and an intention-to-treat (ITT) approach was applied. The final sample consisted of 54 participants (Intervention Group: *n* = 27; Control Group: *n* = 27). Normality of the data distribution was assessed using the Kolmogorov–Smirnov test. Descriptive statistics were used to summarize participants’ baseline characteristics. Continuous variables are presented as means and standard deviations, while categorical variables are reported as absolute and relative frequencies. To examine between-group and longitudinal changes over time, linear mixed models (LMM) were applied. This approach is consistent with the ITT principle and allows for the inclusion of participants with missing data without the need for case-wise exclusion. In the model, group (intervention vs. control), time (pre vs. post), and the group × time interaction were specified as fixed effects, while subject ID was included as a random effect to account for within-subject variability in repeated measurements. Sex and body mass index (BMI) were included as covariates to adjust for potential baseline differences. Post hoc pairwise comparisons were performed using Bonferroni correction. The primary inferential indicator of intervention effectiveness was the group × time interaction effect. Statistical significance was set at *p* ≤ 0.05. Visual inspection of standardized residuals using histograms, Q-Q plots, boxplots, and residual-versus-fitted plots indicated no substantial violations of model assumptions, with a small number of isolated extreme observations identified across outcomes (TUG, ABC). Sensitivity analyses excluding these extreme observations confirmed that the primary results remained unchanged. Specifically, Spearman’s rank-order correlations were computed separately for the Intervention Group and the Control Group across all available time points, rather than on pooled cross-group data. This within-group approach was adopted to avoid methodological confounds inherent in cross-group pooling within an RCT context—namely, between-group variance inflation, differential time effects, and the distortion of correlation coefficients attributable to known group differences—thereby ensuring that reported associations reflect within-group relational patterns only.

## 3. Results

### 3.1. Participant Flow and Baseline Characteristics

**Figure 1:**
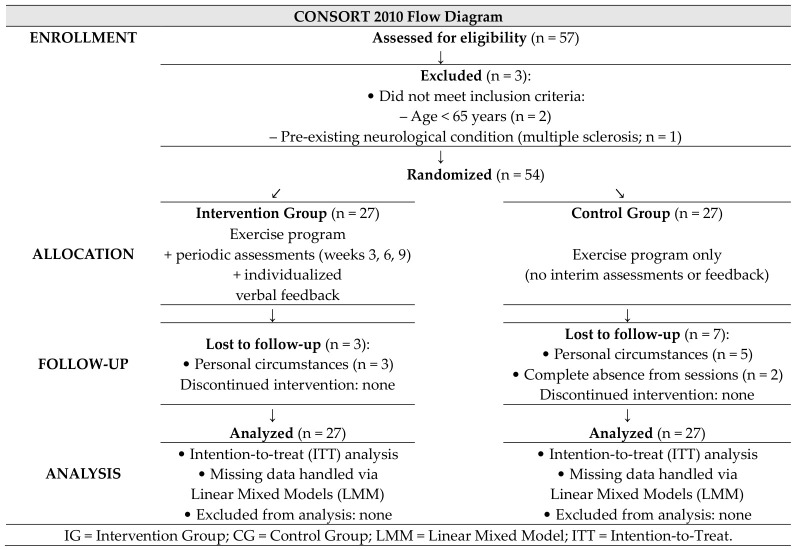
CONSORT diagram of the study.

In total, 57 community-dwelling older adults, registered at municipal senior centers (KAPI) of the Municipality of Nikaia–Agios Ioannis Rentis, were screened for eligibility. Among them, two individuals were excluded due to age (<65 years), and one individual was excluded due to a pre-existing neurological condition (multiple sclerosis). Consequently, 54 adults aged ≥65 years met the inclusion criteria and were enrolled in the study. All eligible participants provided written informed consent prior to participation. After the 12-week period, no adverse events were reported. The CONSORT diagram of the study is detailed in Figure 1, and participant characteristics are presented in Table 2. No statistically significant differences between groups were recorded for most outcome measures at baseline.

### 3.2. Baseline Group Comparisons

Based on Independent samples t-test and non-parametric Mann–Whitney U test analyses for variables following normal and non-normal distributions, respectively, no statistically significant differences were observed between the intervention and control groups in anthropometric characteristics and baseline measurements. Exceptions were observed for exercise self-efficacy (ESE; *p* = 0.013) and exercise motivation (BREQ-2; *p* = 0.035), as shown in Table 3. Overall, these findings indicate a general homogeneity between groups with respect to age, BMI, balance confidence, physical performance, and functional capacity. However, the observed differences in ESE and BREQ-2 suggest partial baseline imbalance in psychological variables, which was accounted for in the subsequent statistical modeling through covariate adjustment.

### 3.3. Group × Time Interaction

Regarding the group × time interaction, which represents the primary indicator of intervention superiority, statistically significant differences were observed for the Short Physical Performance Battery (SPPB; *p* < 0.001), Timed Up and Go test (TUG; *p* = 0.011), and Activities-specific Balance Confidence scale (ABC; *p* < 0.001). These findings indicate significant differential changes over time between the intervention and control groups, in favor of the intervention group. In contrast, no statistically significant group × time interaction effects were found for exercise self-efficacy (ESE) and behavioral regulation in exercise (BREQ-2) (*p* > 0.05), as shown in Table 4.

**Figure 2:**
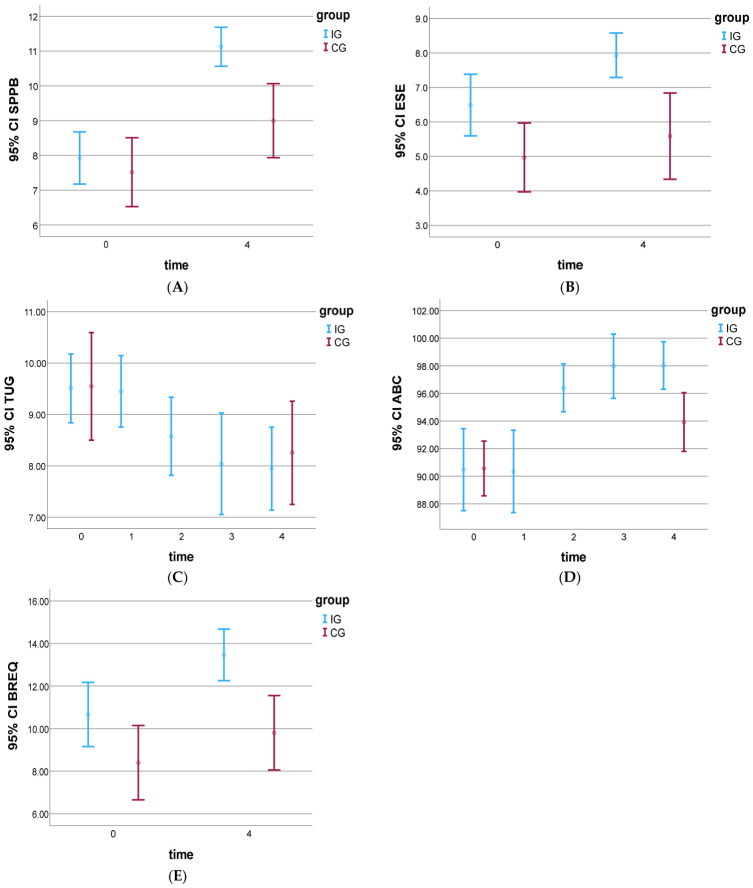
Changes in mean differences (±Standard Error) over time (baseline-0, 3rd week-1, 6th week-2, 9th week-3, final assessment-4) between the Intervention Group (IG) and the Control Group (CG), for the primary and secondary outcome measures: (**A**) Short Physical Performance Battery, (**B**) Self-efficacy for Exercise scale, (**C**) Timed-Up and Go test, (**D**) Activities-specific Balance Confidence scale, (**E**) Behavioral Regulation for Exercise Questionnaire-2.

The following graphs (Figure 2) also present the changes In mean differences (±SE) between the intervention and control groups across the measurement time points (baseline, interim and final assessments) for the primary and secondary outcome measures (SPPB, TUG, ESE, ABC, and BREQ-2).

### 3.4. Post-Intervention Between-Group Comparisons

Statistically significant between-group differences at post-intervention were observed for SPPB (mean difference = 2.078, SE = 0.507, *p* < 0.001; 95% CI: 1.052–3.104), ESE (mean difference = 1.841, SE = 0.626, *p* = 0.006; 95% CI: 0.574–3.108), ABC (mean difference = 3.823, SE = 1.179, *p* = 0.002; 95% CI: 1.438–6.208), and BREQ-2 (mean difference = 3.179, SE = 0.877, *p* < 0.001; 95% CI: 1.416–4.943), all in favor of the intervention group. No statistically significant post-intervention difference was observed for TUG (mean difference = −0.291, SE = 0.580, *p* = 0.619; 95% CI: −1.464–0.883) (Table 5). Notably, post-intervention between-group differences do not constitute primary evidence of intervention superiority in an RCT context; the primary indicator remains the group × time interaction as reported above.

### 3.5. Within-Group Analysis

Within-group analysis revealed statistically significant improvements over time in both groups for SPPB, TUG, and ABC (*p* < 0.05) (Table 6 and Table 7). Regarding psychological outcomes, statistically significant improvements in exercise self-efficacy (ESE) and behavioral regulation in exercise (BREQ-2) were observed only in the intervention group, whereas no significant changes were detected in the control group (*p* > 0.05). These within-group findings are presented as secondary analyses, since in this RCT context, the primary evidence of intervention effect is the group × time interaction.

Regarding the intermediate assessments in the intervention group, statistically significant improvements were observed in the Timed Up and Go test (TUG) between baseline and the second interim assessment (Week 6) (*p* = 0.001), as well as between the second (Week 6) and third interim assessments (Week 9) (*p* = 0.001). No significant difference was observed between the 3rd intermediate assessment (Week 9) and post-intervention (Week 12), indicating a plateau pattern—stabilization following initial improvement—after Week 9. Detailed results for the TUG, including mean differences, standard errors (SE), and 95% confidence intervals (CIs) across all time points, are presented in Table 8.

Similarly, for the Activities-specific Balance Confidence scale (ABC), significant improvements were identified between baseline and the second interim assessment (Week 6) (*p* < 0.001), and between the second (Week 6) and third interim assessments (Week 9) (*p* = 0.001). No significant difference was observed between Week 9 and Week 12. This finding is consistent with a stabilization pattern following the initial response phase. Detailed results for the ABC scale, including mean differences, standard errors (SE), and 95% confidence intervals (CIs) across all time points, are presented in Table 9. It is noted that formal curve modeling was not conducted; therefore, definitive conclusions regarding the optimal timing of peak improvement cannot be drawn.

### 3.6. Spearman’s Correlation Analysis

As most variables did not follow a normal distribution, Spearman’s correlation coefficient was used for within-group analyses. A summary of the statistically significant within-group Spearman’s rank-order correlations by group is presented in Table 10. It is noted that the correlations reported below were initially computed on pooled data across both groups and time points. To address the methodological limitations of this approach—including between-group variance inflation and differential time effects—Spearman’s rank-order correlations were subsequently recomputed separately for the Intervention Group and the Control Group (Table 11 and Table 12). All findings reported hereafter refer to these within-group analyses. These correlations are exploratory in nature, were computed across multiple time points within each group without formal adjustment for repeated measures or temporal autocorrelation and do not imply causal relationships. 

**Table 10:** Summary of statistically significant within-group Spearman’s rank-order correlations by group.

Variable Pair	IG: ρs (*p*)	CG: ρs (*p*)
SPPB & ESE	0.400 (0.004); Moderate (+)	0.439 (0.002); Moderate (+)
SPPB & BREQ-2	0.485 (<0.001); Moderate (+)	—
SPPB & TUG	−0.668 (<0.001); Strong (−)	−0.876 (<0.001); Strong (−)
SPPB & ABC	0.761 (<0.001); Strong (+)	0.764 (<0.001); Strong (+)
ESE & BREQ-2	0.444 (0.001); Moderate (+)	0.350 (0.017); Moderate (+)
ESE & TUG	−0.454 (<0.001); Moderate (−)	−0.353 (0.016); Moderate (−)
ESE & ABC	0.448 (<0.001); Moderate (+)	—
ESE & Attendance rate	0.394 (0.004); Moderate (+)	—
BREQ-2 & ABC	0.355 (0.011); Moderate (+)	—
BREQ-2 & Attendance rate	—	0.398 (0.006); Moderate (+)
TUG & ABC	−0.650 (<0.001); Strong (−)	−0.681 (<0.001); Strong (−)
TUG & Attendance rate	−0.226 (0.013); Weak (−)	—
ABC & Attendance rate	0.195 (0.034); Weak (+)	—

SPPB: Short Physical Performance Battery; TUG: Timed-Up and Go test; ESE: Exercise Self-Efficacy scale; ABC: Activities-specific Balance Confidence scale; BREQ-2: Behavioral Regulation for Exercise Questionnaire-2.

**Table 11:** Spearman’s correlation analysis for the intervention group.

Spearman’s Rho		SPPB	ESE	BREQ	TUG	ABC	Attendance Rate (%)
SPPB	Correlation Coefficient	1.000	0.400 **	0.485 **	−0.668 **	0.761 **	0.265
	Sig. (2-tailed)		0.004	<0.001	<0.001	<0.001	0.060
ESE	Correlation Coefficient	0.400 **	1.000	0.444 **	−0.454 **	0.448 **	0.394 **
	Sig. (2-tailed)	0.004		0.001	<0.001	<0.001	0.004
BREQ-2	Correlation Coefficient	0.485 **	0.444 **	1.000	−0.217	0.355 *	0.093
	Sig. (2-tailed)	<0.001	0.001		0.126	0.011	0.515
TUG	Correlation Coefficient	−0.668 **	−0.454 **	−0.217	1.000	−0.650 **	−0.226 *
	Sig. (2-tailed)	<0.001	<0.001	0.126		<0.001	0.013
ABC	Correlation Coefficient	0.761 **	0.448 **	0.355 *	−0.650 **	1.000	0.195 *
	Sig. (2-tailed)	<0.001	<0.001	0.011	<0.001		0.034
Attendance rate (%)	Correlation Coefficient	0.265	0.394 **	0.093	−0.226 *	0.195 *	1.000
	Sig. (2-tailed)	0.060	0.004	0.515	0.013	0.034	

SPPB: Short Physical Performance Battery; TUG: Timed-Up and Go test; ESE: Exercise Self-Efficacy scale; ABC: Activities-specific Balance Confidence scale; BREQ-2: Behavioral Regulation for Exercise Questionnaire-2. Correlations were considered statistically significant at ** *p* < 0.01 and * *p* < 0.05.

**Table 12:** Spearman’s correlation analysis for the control group.

Spearman’s Rho		SPPB	ESE	BREQ	TUG	ABC	Attendance Rate (%)
SPPB	Correlation Coefficient	1.000	0.439 **	0.236	−0.876 **	0.764 **	0.066
	Sig. (2-tailed)		0.002	0.115	<0.001	<0.001	0.665
ESE	Correlation Coefficient	0.439 **	1.000	0.350 *	−0.353 *	0.264	0.265
	Sig. (2-tailed)	0.002		0.017	0.016	0.076	0.075
BREQ-2	Correlation Coefficient	0.236	0.350 *	1.000	−0.256	0.078	0.398 **
	Sig. (2-tailed)	0.115	0.017		0.086	0.608	0.006
TUG	Correlation Coefficient	−0.876 **	−0.353 *	−0.256	1.000	−0.681 **	−0.062
	Sig. (2-tailed)	<0.001	0.016	0.086		<0.001	0.684
ABC	Correlation Coefficient	0.764 **	0.264	0.078	−0.681 **	1.000	−0.030
	Sig. (2-tailed)	<0.001	0.076	0.608	<0.001		0.845
Attendance rate (%)	Correlation Coefficient	0.066	0.265	0.398 **	−0.062	−0.030	1.000
	Sig. (2-tailed)	0.665	0.075	0.006	0.684	0.845	

SPPB: Short Physical Performance Battery; TUG: Timed-Up and Go test; ESE: Exercise Self-Efficacy scale; ABC: Activities-specific Balance Confidence scale; BREQ-2: Behavioral Regulation for Exercise Questionnaire-2. Correlations were considered statistically significant at ** *p* < 0.01 and * *p* < 0.05.

#### 3.6.1. Intervention Group

Physical performance and functional capacity, as assessed by the Short Physical Performance Battery (SPPB), were moderately positively correlated with exercise self-efficacy (ESE; ρ = 0.400, *p* = 0.004) and behavioral regulation in exercise (BREQ-2; ρ = 0.485, *p* < 0.001), and strongly positively correlated with balance confidence (ABC; ρ = 0.761, *p* < 0.001). SPPB demonstrated a strong negative correlation with the Timed Up and Go test (TUG; ρ = −0.668, *p* < 0.001). Similarly, moderate positive correlations were observed between ESE and BREQ-2 (ρ = 0.444, *p* = 0.001), as well as between ESE and ABC (ρ = 0.448, *p* < 0.001). Finally, exercise adherence -expressed as attendance rate- demonstrated a moderate positive correlation with exercise self-efficacy (ρ = 0.394, *p* = 0.004), a low positive correlation with balance confidence (ABC; ρ = 0.195, *p* = 0.034), as well as a low negative correlation with TUG (ρ = −0.226, *p* = 0.013), as presented in Table 11.

#### 3.6.2. Control Group

Physical performance and functional capacity, as assessed by the Short Physical Performance Battery (SPPB), were moderately positively correlated with exercise self-efficacy (ESE; ρ = 0.439, *p* = 0.002) and strongly positively correlated with balance confidence (ABC; ρ = 0.764, *p* < 0.001). SPPB demonstrated a strong negative correlation with the Timed Up and Go test (TUG; ρ = −0.876, *p* < 0.001) and no correlation with behavioral regulation in exercise (BREQ-2; ρ = 0.236, *p* = 0.115). Exercise adherence -expressed as attendance rate- demonstrated a moderate positive correlation with BREQ-2 (ρ = 0.398, *p* = 0.006) and no correlation with any other outcome measures, as presented in Table 12.

### 3.7. Participants’ Exercise Adherence

Based on attendance records maintained throughout the intervention period, the intervention group demonstrated a mean attendance of 13.41 sessions (±4.67), corresponding to 55.9% of the prescribed sessions. This reflects a moderate level of adherence. In the control group, mean attendance was 10.93 sessions (±5.74), corresponding to 45.54% of the total prescribed sessions, indicating a numerically and statistically significant (*p* = 0.006) lower adherence compared to the intervention group, as shown in Table 13. Notably, two participants in the control group attended no sessions, while two additional participants attended only one session. Both values fall below the ≥80% threshold considered satisfactory in the literature [57,58].

## 4. Discussion

### 4.1. Principal Findings

The present pilot RCT suggests that periodic assessments followed by encouraging verbal feedback, in conjunction with a structured exercise program in healthy community-dwelling older adults, may represent a valuable adjunct for improving selected functional outcomes of exercise interventions. Importantly, the primary composite hypothesis was only partially supported; while statistically significant group × time interactions were identified for the primary functional outcome (SPPB) and the secondary functional outcomes (TUG, ABC), no significant between-group effect was observed for the primary psychological outcome, exercise self-efficacy (ESE; *p* = 0.108). Findings should therefore be interpreted in light of this distinction between primary and secondary outcomes and should be considered preliminary and hypothesis-generating rather than confirmatory, given the pilot nature of the study and the absence of an a priori sample size calculation.

The primary finding of the study relates to the significant group × time interaction observed for selected outcome measures (SPPB, TUG, and ABC), indicating differential trajectories of improvement between the two groups. Specifically, statistically significant interaction effects were identified for physical performance (SPPB; *p* < 0.001), functional mobility (TUG; *p* = 0.011), and balance confidence (ABC; *p* < 0.001), suggesting that the intervention group exhibited a more favorable trajectory of functional improvement than the control group over the intervention period. Importantly, the primary hypothesis was only partially supported, as significant between-group effects over time were not observed in all predefined primary outcomes. While improvements were observed in physical performance as measured by the SPPB, no statistically significant group × time interaction was found for exercise self-efficacy (ESE). In contrast, statistically significant effects were identified in secondary outcomes, particularly in functional measures, such as functional mobility, as assessed by the TUG test, and balance confidence, as assessed by the ABC scale. This pattern indicates that the overall effect of the intervention was primarily driven by changes in functional outcomes, rather than psychological outcomes and self-efficacy, and may reflect the contribution of periodic assessments and verbal feedback as mechanisms that may enhance responsiveness to exercise interventions. However, given the pilot nature of the study and the relatively small sample size, these findings should be interpreted with caution and require confirmation in larger-scale RCTs before causal inferences can be established. Of particular note, the TUG test showed a significant group × time interaction (TUG; *p* = 0.011), yet the post-intervention between-group comparison was not statistically significant (TUG; *p* = 0.619, Table 5). This pattern may indicate that the intervention group experienced a more rapid improvement during the intervention period, whereas the control group reached a comparable absolute level by Week 12. However, given the pilot design and absence of an a priori power calculation, this interpretation should be considered exploratory, and the lack of a statistically significant post-intervention between-group difference may also reflect limited statistical power. Differences between change trajectories and endpoint values should therefore be interpreted cautiously when evaluating the intervention’s effects on functional mobility.

In contrast, no significant group × time interaction effects were observed for exercise self-efficacy (ESE) and exercise motivation (BREQ-2), indicating that the rate of change during time in these psychological parameters did not differ significantly between groups. This may be attributed either to improvements occurring in both groups due to the common exercise program or to a potentially limited statistical power to detect changes in psychological outcomes. It is also important to note that statistically significant post-intervention differences observed in ESE (*p* = 0.006) and BREQ-2 (*p* < 0.001) do not constitute primary evidence of intervention superiority, as the group × time interaction—considered the main indicator of effectiveness—was not significant for these variables (*p* = 0.108 and *p* = 0.164, respectively). Additionally, it should be acknowledged that statistically significant baseline differences between groups were observed for ESE (*p* = 0.013) and BREQ-2 (*p* = 0.035) in favor of the IG (Table 3). Although these were addressed through covariate adjustment in the LMM, the pre-existing psychological advantage of the IG should be considered when interpreting post-intervention between-group differences in ESE (*p* = 0.006) and BREQ-2 (*p* < 0.001), as these may partially reflect this initial imbalance rather than solely the effect of the intervention. These findings indicate that the intervention led to improvements primarily in functional performance, with a more limited impact on psychological outcomes.

Although the observed interaction effects remained robust in sensitivity analyses excluding extreme residual observations, the absence of an a priori sample size calculation precludes definitive conclusions regarding statistical power. Consequently, both significant and non-significant findings should be interpreted as preliminary estimates intended to inform the design and sample size calculation of future adequately powered randomized controlled trials.

### 4.2. Functional Outcomes

Within-group improvements were observed in both groups across all outcome measures, including physical performance and functional capacity, exercise motivation, self-efficacy, and balance confidence. These changes likely reflect the potential effects associated with participation in a structured exercise program. Notably, the within-group improvement in the IG for SPPB reached 3.151 points (Table 6), representing a notable within-group change; however, this should be interpreted cautiously in the absence of between-group adjustment and prespecified clinical threshold analyses. Similarly, the within-group TUG improvement in the IG (1.573 s) exceeded that of the CG (0.849 s), with the between-group difference of approximately 1.3 s, suggesting a greater magnitude of change in the IG, although no definitive conclusions regarding clinical relevance can be drawn without predefined MCID-based analyses. These findings are consistent with previous literature reporting beneficial effects of resistance and balance training on physical performance and functional capacity in community-dwelling older adults [59,60,61,62,63,64].

The effect of the structured strengthening component of the exercise intervention is supported by evidence showing that resistance training in older adults is associated with improvements in neuromuscular function through enhanced motor unit recruitment, increased voluntary activation, and improved neuromuscular efficiency; adaptations that collectively may contribute to enhanced force production in the lower limbs [6,65]. Such neuromuscular improvements are functionally relevant for tasks such as sit-to-stand and gait initiation, which are key determinants of performance in mobility-based assessments, including the SPPB and TUG tests. In parallel, the repetitive and task-specific nature of the balance component of the exercise program is consistent with evidence indicating that repeated practice of functional and postural tasks may induce motor learning–related adaptations, leading to improved postural control and reduced sway during dynamic conditions [66,67]. Task-specific and balance training interventions have been shown to improve mobility and balance performance in older adults, likely through enhanced integration of sensory feedback and more efficient motor planning and execution processes [68,69].

From a mechanistic standpoint, these adaptations reflect the combined effects of neuromuscular and sensorimotor plasticity, whereby repeated functional exposure enhances intermuscular coordination, movement efficiency, and the integration of proprioceptive, visual, and vestibular inputs within the central nervous system [6,70]. Multicomponent exercise programs combining strength, balance and coordination elements have been shown to improve functional performance and are associated with biological markers linked to neuroplasticity, such as epigenetic modulation and neurotrophic factors [70]. Repeated exposure to functional, task-specific activities further supports motor learning processes, leading to more efficient movement strategies and improved intermuscular coordination [20]. Collectively, these neurophysiological adaptations are more directly reflected in objective, performance-based outcome measures, such as the Timed Up and Go test and balance assessments, which capture changes in functional capacity and movement efficiency [71]. In contrast, self-reported constructs, such as exercise self-efficacy, are influenced by a broader range of psychological and contextual factors and may not exhibit parallel changes over short intervention periods, as described within the framework of Social Cognitive Theory [10,14].

The effect of a behavioral intervention based on Albert Bandura’s Social Cognitive Theory (1997), combined with an exercise program, was investigated in the study of Larsen et al. (2021) [72], with results showing a clinically relevant, but not statistically significant, difference in favor of the intervention group in terms of physical activity. In a contrasting direction, unlike Larsen et al. (2021), who found no statistically significant between-group effect on the primary outcome of daily step count despite a clinically relevant difference, the present study identified significant group × time interactions for functional performance outcomes (SPPB, TUG, ABC). However, the substantial differences in study design, intervention type, outcome measures, and population between the two studies preclude direct comparison. In the 9th week of the exercise program, statistically significant differences in TUG and ABC were also observed compared with the 6th week assessment in the IG, whereas a plateau pattern was noted between the 9th week and the final evaluation (week 12). It should be noted that this pattern pertains specifically to TUG and ABC, which were the only outcomes assessed at interim time points; SPPB was not measured at Weeks 3, 6, or 9, and therefore no equivalent temporal trajectory can be reported for this measure. This pattern suggests an initial improvement at mid-intervention, followed by a subsequent phase of outcome stabilization, consistent with a plateau effect, i.e., stabilization of outcomes after an initial improvement. However, it should be noted that robust conclusions regarding the timing of peak improvement would require curve modeling approaches, which were not applied in the present study. Furthermore, no statistically significant improvement was observed between baseline and the first interim assessment (Week 3) for either TUG (*p* = 1.000, Table 8) or ABC (*p* = 1.000, Table 9), suggesting that measurable functional gains were first detected after six weeks in the present sample. Whether this reflects a true temporal threshold or is influenced by sampling variability and limited statistical power cannot be determined from the present pilot study. This finding might have practical implications for the timing of reassessment in future exercise programs targeting functional mobility and balance confidence in community-dwelling older adults.

The addition of periodic assessments and verbal feedback may have contributed to the observed effects, potentially by enhancing sensorimotor integration and movement accuracy. Feedback may have supported error detection and correction, reinforced optimal movement strategies, and increased participants’ confidence in task execution. Motor-cognitive exercise with augmented feedback and variable practice conditions has been shown to improve functional ability and cognitive performance, suggesting a potential role for feedback-enriched training in motor learning and neural adaptation processes in older adults [73]. Additionally, interactive and technology-assisted multimodal interventions (e.g., augmented reality or sensor-based training) have been associated with improvements in executive function, reaction time, and dual-task performance, which may reflect enhanced sensorimotor integration and central processing efficiency [74]. Collectively, these findings are consistent with the hypothesis that combining multimodal exercise with structured feedback mechanisms may be associated with neuroplastic adaptations and improve the integration of sensory and motor information within the central nervous system in aging populations.

Previous studies in healthy older adults support the notion that improvements in psychological and functional outcomes following exercise typically emerge after several weeks of exposure to the intervention, while continued participation contributes to the stabilization and long-term maintenance of these effects [4,75]. In the same direction, the maintenance of long-term outcomes appears to require the use of structured motivational strategies that enhance adherence and support sustained health-related behavior change [76]. These findings align with existing literature suggesting that multimodal exercise interventions, especially those incorporating feedback, can potentiate neuroplastic changes and improve the efficiency of sensorimotor processing in older adults [77].

### 4.3. Psychological Outcomes

The findings of the present study indicate that individualized verbal feedback based on periodic assessments may be associated with enhanced self-confidence in older adults, as suggested by the significant post-intervention between-group difference in ESE (*p* = 0.006). However, this interpretation should be made with caution, as this finding reflects a post-intervention point-in-time comparison and does not constitute primary evidence of intervention superiority; the group × time interaction for ESE was not statistically significant (*p* = 0.108), limiting the strength of this inference. This further supports the role of behavioral approaches, as also investigated in studies, such as those by Yeom & Fleury (2014) [78] and Fang et al. (2024) [76], in which interventions based on the Wellness Motivation Theory (WMT) were applied [79]. Specifically, Yeom et al. (2014) reported that older adults who received a motivational intervention for physical activity demonstrated significant improvements in behavioral change processes, levels of physical activity, and mobility outcomes, findings that are consistent with Fang et al. (2024), where continuous supervision and guidance were found to enhance participants’ self-confidence and adherence to exercise.

However, there was no statistically significant group × time interaction for behavioral outcomes -ESE and BREQ-2- in the present study; this finding may reflect either similar improvement in both groups attributable to the shared exercise program, or insufficient statistical power to detect differences in these psychological constructs in a pilot-sized sample. It is noteworthy that within-group analysis revealed no statistically significant improvements in ESE (*p* = 0.564) or BREQ-2 (*p* = 0.280) in the CG over the 12-week period (Table 7), in contrast to the IG, where both outcomes improved significantly (*p* = 0.001 and *p* < 0.001, respectively, Table 6). While this pattern does not constitute confirmatory evidence of intervention superiority in the absence of a significant group × time interaction, it may suggest a differential psychological response to exercise participation depending on the presence of structured feedback, warranting further investigation in adequately powered trials. Alternative explanations cannot be ruled out, and these findings highlight the need for future fully powered studies with adequate sample sizes to address this question. Overall, the intervention demonstrates efficacy primarily in functional domains, and interpretation of its effectiveness should be framed accordingly, acknowledging that the primary hypothesis was only partially confirmed and that functional outcomes were the main drivers of the observed effects.

Within-group correlational analyses further illuminate the differential impact of the intervention on the relationship between psychological and functional constructs. In the IG, a broader and more interconnected pattern of associations was observed across functional, psychological, and adherence-related variables compared to the CG. Notably, ESE was significantly associated not only with functional performance (SPPB, TUG, ABC) but also with exercise motivation (BREQ) and attendance in the IG, whereas several of these associations were absent or attenuated in the CG. Specifically, while ESE remained significantly associated with SPPB (ρ = 0.439, *p* = 0.002), TUG (ρ = −0.353, *p* = 0.016), and BREQ-2 (ρ = 0.350, *p* = 0.017) in the CG, the associations between ESE and ABC (*p* = 0.076) and between ESE and attendance (*p* = 0.075) did not reach statistical significance, suggesting a more limited role of self-efficacy as a correlate of balance confidence and exercise participation in the absence of feedback. This pattern is consistent with the theoretical framework of Bandura’s Social Cognitive Theory (1997), which posits that enhanced self-efficacy reinforces motivational processes and sustains health-related behaviors—a dynamic that may have been activated through the periodic assessment and verbal feedback received by the IG. The stronger SEE–attendance association observed in the IG (rs = 0.394, *p* = 0.004) relative to the CG (rs = 0.265, *p* = 0.075) suggests that the intervention may have potentiated the role of perceived competence as a driver of exercise participation, supporting the practical utility of feedback-based strategies for promoting adherence in older adults.

It should be noted, however, that these correlational findings are exploratory in nature and were computed across multiple time points within each group, without formal adjustment for repeated measures or temporal autocorrelation. As such, they should be interpreted cautiously and regarded as hypothesis-generating rather than confirmatory. Future studies employing multilevel modeling or cross-lagged panel approaches would be better positioned to establish the directionality and temporal stability of these relationships.

### 4.4. Exercise Adherence

With respect to exercise adherence, the levels observed in the present study were suboptimal. The IG’s attendance rate was 55.9% of the total prescribed sessions (24 sessions), while the CG’s attendance rate was 45.54%, both falling well below the ≥80% threshold commonly considered indicative of satisfactory adherence in exercise interventions [57,58]. This finding aligns with existing concerns in the literature regarding low participation and adherence rates in exercise programs among older adults, as highlighted in systematic reviews involving both healthy individuals and those with chronic conditions [4,58,75].

A similar pattern of findings has been reported in studies incorporating follow-ups and continuous monitoring of participation, such as the study by Rikkonen et al. (2023) [80], who reported adherence rates of 70.4–79.9% in an IG receiving biweekly telephone-based contact in addition to the exercise program over 24 months; however, this comparison should be interpreted with caution, considering the substantial methodological differences between the two studies, including intervention duration (24 months vs. 12 weeks), the nature of the adherence-enhancing strategy (biweekly telephone contact vs. in-person periodic assessment with verbal feedback), participant population, and outcome assessment methods. These differences preclude meaningful direct comparison of adherence rates across studies.

The overall exercise participation rate in the intervention group, which received three additional interim assessments and reinforcement through verbal feedback, reached 55.9%, exceeding that of the control group (45.54%) by approximately 10.36 percentage points. These findings indicate moderate overall adherence to the exercise program among participants, alongside a numerically and statistically significant higher participation rate in the intervention group (*p* = 0.006). This finding, along with the observed low to moderate correlations between exercise adherence and exercise self-efficacy (ESE; ρ = 0.394, *p* = 0.004), balance confidence (ABC; ρ = 0.195, *p* = 0.034) and functional mobility (TUG; ρ = −0.226, *p* = 0.013) in the intervention group only, suggest an association between higher self-efficacy, better balance confidence, and greater exercise participation specifically within the intervention context. These correlational findings are exploratory in nature and cannot establish causality or directionality; however, they are consistent with the hypothesis that periodic assessment and verbal feedback may support adherence by reinforcing perceived competence and functional confidence.

### 4.5. Limitations

The present pilot study shows several limitations. Given the pilot nature of the study and the limited sample size, all findings should be interpreted as preliminary and hypothesis-generating rather than confirmatory, thereby limiting the direct generalizability to the broader population of community-dwelling older adults. As the final sample was effectively female-only, the generalizability of the present findings is limited to community-dwelling older women, and results should not be extrapolated to the broader older adult population of both sexes without further investigation.

The relatively low adherence rates to the exercise program, with participants attending on average approximately half of the scheduled sessions, constitute an additional important limitation; this may have led to a reduced effect size and the inability to detect statistically significant differences. Low adherence may have also led to a potential attenuation of the exercise intervention effect, as participants were not exposed to the full “dose” of the intended stimulus, since in exercise-based interventions, the magnitude of improvement in physical, functional, and psychological outcomes is closely related to the frequency and consistency of participation. Lastly, low adherence may have acted as a potential mediator of the intervention effects on the outcome measures, although no formal mediation analysis was conducted. Despite the low adherence rates, the difference between groups was statistically significant (*p* = 0.006), and statistically significant improvements were still observed in outcome measures, both for the group × time interaction and within-group comparisons. This finding, however, raises important considerations regarding potential mediating mechanisms and highlights the need for future studies to explore strategies that could enhance adherence and, consequently, maximize exercise-related benefits. A per-protocol analysis was considered as a sensitivity approach; however, only three participants in the intervention group and none in the control group achieved the predefined adherence threshold (≥80%). Therefore, a per-protocol analysis was not statistically feasible due to extreme group imbalance and insufficient sample size, and the intention-to-treat analysis was retained as the primary analytical approach.

Another limitation of the study relates to its methodological design, particularly the absence of assessor blinding and the unequal amount of participant–researcher contact between groups. The same physiotherapist conducted all outcome assessments and delivered individualized verbal feedback to participants in the intervention group while being aware of group allocation. Although standardized assessment procedures were followed, the possibility of assessor bias cannot be excluded, especially for performance-based measures such as the SPPB and TUG, where subtle differences in instruction, encouragement, or interpretation may influence participants’ performance. Likewise, the ABC scale, although self-reported, may have been indirectly affected by the interaction established with the assessor.

Furthermore, participants in the intervention group underwent three additional assessments and received more frequent contact with the assessor than those in the control group. This increased attention may have heightened participants’ awareness of being observed and monitored, which may have led to enhanced motivation, engagement, confidence, or adherence independently of the exercise intervention itself, reflecting a potential Hawthorne effect [81]. Consequently, part of the observed group × time interactions for SPPB, TUG, and ABC may have been influenced not only by feedback-based intervention but also by increased researcher attention and the lack of assessor blinding. Therefore, the magnitude of the intervention effect should be interpreted with caution. Future randomized controlled trials should incorporate blinded outcome assessors and equalize participant–researcher contact across groups to minimize both performance and detection bias.

Finally, no statistically significant group × time interaction was observed for the psychological variables ESE and BREQ-2, which may be partially explained by insufficient statistical power to detect differences in these outcomes. The presence of missing data in the final assessments represents an additional limitation; however, appropriate statistical models were used to handle missing values in order to minimize the risk of systematic bias.

## 5. Conclusions

The present pilot RCT provides partial support for the effectiveness of systematic assessments followed by individualized verbal feedback as an adjunct to a structured exercise program in healthy community-dwelling adults-mostly women-aged ≥65 years. The primary composite hypothesis was only partially confirmed; significant group × time interactions were observed for one of the primary outcomes (SPPB: *p* < 0.001) and secondary functional outcomes (TUG: *p* = 0.011; ABC: *p* < 0.001), particularly with regard to physical fitness, functional capacity, and balance confidence, whereas no significant group × time interaction was found for the primary psychological outcome, ESE (*p* = 0.108), or for BREQ-2 (*p* = 0.164), possibly reflecting insufficient statistical power to detect between-group differences in psychological constructs in a pilot-sized sample. Overall, the intervention demonstrates efficacy primarily in functional domains, and its effectiveness should be interpreted accordingly. Interpretation of the findings related to ESE and BREQ-2 should also be made with caution due to significant baseline differences between groups that favored the intervention group, despite statistical adjustment, which may have influenced post-intervention comparisons.

Despite its positive findings, the study presents several limitations that should be considered when interpreting the results and drawing firm conclusions. Its pilot nature, the limited sample size with a predominantly female representation, the potential influence of the Hawthorne effect, and the presence of missing data all restrict the generalizability and the strength of the inferences that can be made. Moderate overall adherence in both groups may also have acted as a potential mediating factor, warranting formal mediation analysis in future studies. To support the design of a future definitive randomized controlled trial, effect size estimates derived from the present pilot data should be used to inform a priori power calculations, thereby ensuring adequate statistical power to detect clinically meaningful between-group differences in both functional and psychological outcomes. Future fully powered RCTs with larger, representative samples, long-term follow-up, sex-balanced enrollment, and integrated adherence-enhancement strategies are needed to confirm the findings of the present study in order to optimize the frequency and content of feedback protocols during exercise interventions for the geriatric population.

## 6. Ethical Statement

This study was conducted in full compliance with the ethical principles governing scientific research, in accordance with the Declaration of Helsinki (World Medical Association, 2013) [29]. Approval was obtained from the Ethics and Research Committee (E.H.D.E.) of the University of West Attica, Greece (protocol No. 107620/24-10-2025). All participants provided written informed consent. Participation was voluntary and uncompensated. Participant data were anonymized and managed in accordance with the General Data Protection Regulation (GDPR) and applicable Greek legislation.

## Figures and Tables

**Table 1:** Assessment timing.

Outcome Measure	Baseline	Week 3	Week 6	Week 9	Week 12
SPPB	IG & CG	-	-	-	IG & CG
ESE	IG & CG	-	-	-	IG & CG
TUG	IG & CG	IG	IG	IG	IG & CG
ABC	IG & CG	IG	IG	IG	IG & CG
BREQ-2	IG & CG	-	-	-	IG & CG

**Table 2:** Participants’ characteristics.

Participants’ Characteristics	Sample (Ν = 54)	Intervention Group (N = 27)	Control Group (N = 27)
Sex			
Men	1	-	1
Women	53	26	26
Age (years; mean, SD)	74.76 (6.23)	73.56 (6.54)	75.96 (5.76)
BMI (kg/m^2^; mean, SD)	28.45 (4.26)	27.84 (4.26)	29.06 (4.26)
Prior participation in exercise (*n*)			
Yes	32	18	14
No	22	9	13
Smoking (*n*)			
Yes	12	6	6
No	42	21	21
Number of falls in the last 12 months (*n*)			
No falls	28	19	9
One fall	19	7	12
More than one fall	7	1	6

SD: standard deviation; BMI: body mass index.

**Table 3:** Baseline outcome measures—between-group comparisons (LMM).

Variable	Mean Difference IG-CG	Standard Error	*p*-Value	95% CI Lower Bound	95% CI Upper Bound
SPPB	0.571	0.602	0.348	−0.639	1.781
TUG	−0.194	0.608	0.752	−1.415	1.028
ESE	1.653	0.644	0.013	0.359	2.947
ABC	−0.193	1.744	0.913	−3.695	3.310
BREQ-2	2.471	1.138	0.035	0.186	4.757

SPPB: Short Physical Performance Battery; TUG: Timed-Up and Go test; ESE: Exercise Self-Efficacy scale; ABC: Activities-specific Balance Confidence scale; BREQ-2: Behavioral Regulation for Exercise Questionnaire-2; IG: Intervention Group; CG: Control Group; CI: Confidence Interval.

**Table 4:** Group × Time Interaction *p*-values for primary and secondary outcome measures.

Variable	*p*-Value (Group × Time)
SPPB	<0.001
TUG	0.011
ESE	0.108
ABC	<0.001
BREQ-2	0.164

SPPB: Short Physical Performance Battery; TUG: Timed-Up and Go test; ESE: Exercise Self-Efficacy scale; ABC: Activities-specific Balance Confidence scale; BREQ-2: Behavioral Regulation for Exercise Questionnaire-2.

**Table 5:** Post-intervention outcome measures’ comparisons between groups.

Variable	Mean Difference (IG-CG)	Standard Error	*p*-Value	95% CI Lower Bound	95% CI Upper Bound
SPPB	2.078	0.507	<0.001	1.052	3.104
TUG	−0.291	0.580	0.619	−1.464	0.883
ESE	1.841	0.626	0.006	0.574	3.108
ABC	3.823	1.179	0.002	1.438	6.208
BREQ-2	3.179	0.877	<0.001	1.416	4.943

SPPB: Short Physical Performance Battery; TUG: Timed-Up and Go test; ESE: Exercise Self-Efficacy scale; ABC: Activities-specific Balance Confidence scale; BREQ-2: Behavioral Regulation for Exercise Questionnaire-2; IG: Intervention Group; CG: Control Group; CI: Confidence Interval.

**Table 6:** Within-group pre–post comparison: Intervention Group (IG).

Variable	Mean Difference	Standard Error	df	*p*-Value	95% CI Lower Bound	95% CI Upper Bound
SPPΒ	3.151	0.280	24.344	<0.001	2.574	3.728
TUG	−1.573	0.182	88.388	<0.001	−2.097	−1.049
ESE	1.467	0.403	22.050	0.001	0.633	2.302
ABC	7.375	0.708	88.169	<0.001	5.337	9.413
BREQ-2	3.009	0.749	23.396	<0.001	1.146	4.558

SPPB: Short Physical Performance Battery; TUG: Timed-Up and Go test; ESE: Exercise Self-Efficacy scale; ABC: Activities-specific Balance Confidence scale; BREQ-2: Behavioral Regulation for Exercise Questionnaire-2; IG: Intervention Group; CG: Control Group; CI: Confidence Interval.

**Table 7:** Within-group pre–post comparison: Control Group (CG).

Variable	Mean Difference	Standard Error	df	*p*-Value	95% CI Lower Bound	95% CI Upper Bound
SPPΒ	1.124	0.317	18.619	0.002	0.460	1.789
TUG	−0.849	0.201	18.071	<0.001	−1.27	−0.428
ESE	0.341	0.581	18.751	0.564	−0.875	1.558
ABC	3.507	0.446	18.180	<0.001	2.570	4.443
BREQ-2	1.209	1.088	19.660	0.280	−1.063	3.480

SPPB: Short Physical Performance Battery; TUG: Timed-Up and Go test; ESE: Exercise Self-Efficacy scale; ABC: Activities-specific Balance Confidence scale; BREQ-2: Behavioral Regulation for Exercise Questionnaire-2; IG: Intervention Group; CG: Control Group; CI: Confidence Interval.

**Table 8:** Within-group comparisons in the intervention group (IG) across baseline (pre), interim, and post-intervention assessments for the Timed Up and Go test (TUG).

Timeline		Mean Difference	Standard Error	df	*p*-Value	95% Confidence Interval	
						Lower Bound	Upper Bound
0 (baseline)	1	0.057	0.177	88.279	1.000	−0.454	0.567
	2	0.788 *	0.187	88.344	0.001	0.250	1.325
	3	1.665 *	0.193	88.523	0.000	1.108	2.222
	4	1.573 *	0.182	88.388	0.000	1.049	2.097
1st (Week 3)	0	−0.057	0.177	88.279	1.000	−0.567	0.454
	2	0.731 *	0.189	88.498	0.002	0.186	1.277
	3	1.609 *	0.194	88.420	0.000	1.049	2.168
	4	1.516 *	0.183	88.278	0.000	0.990	2.043
2nd (Week 6)	0	−0.788 *	0.187	88.344	0.001	−1.325	−0.250
	1	−0.731 *	0.189	88.498	0.002	−1.277	−0.186
	3	0.877 *	0.206	88.821	0.001	0.283	1.472
	4	0.785 *	0.195	88.645	0.001	0.225	1.345
3rd (Week 9)	0	−1.665 *	0.193	88.523	0.000	−2.222	−1.108
	1	−1.609 *	0.194	88.420	0.000	−2.168	−1.049
	2	−0.877 *	0.206	88.821	0.001	−1.472	−0.283
	4	−0.092	0.196	88.313	1.000	−0.658	0.473
4th (Week 12)	0	−1.573 *	0.182	88.388	0.000	−2.097	−1.049
	1	−1.516 *	0.183	88.278	0.000	−2.043	−0.990
	2	−0.785 *	0.195	88.645	0.001	−1.345	−0.225
	3	0.092	0.196	88.313	1.000	−0.473	0.658

* Level of statistical significance: *p* < 0.05.

**Table 9:** Within-group comparisons in the intervention group (IG) across baseline (pre), interim, and post-intervention assessments for the Activities-specific Balance Confidence scale (ABC).

Timeline		Mean Difference	Standard Error	df	*p*-Value	95% Confidence Interval	
						Lower Bound	Upper Bound
0 (baseline)	1	−0.014	0.689	88.025	1.000	−1.997	1.970
	2	−5.046 *	0.726	88.115	0.000	−7.137	−2.956
	3	−7.547 *	0.752	88.351	0.000	−9.713	−5.382
	4	−7.375 *	0.708	88.169	0.000	−9.413	−5.337
1st (Week 3)	0	0.014	0.689	88.025	1.000	−1.970	1.997
	2	−5.033 *	0.736	88.316	0.000	−7.153	−2.912
	3	−7.534 *	0.755	88.216	0.000	−9.708	−5.359
	4	−7.362 *	0.711	88.025	0.000	−9.409	−5.314
2nd (Week 6)	0	5.046 *	0.726	88.115	0.000	2.956	7.137
	1	5.033 *	0.736	88.316	0.000	2.912	7.153
	3	−2.501 *	0.802	88.747	0.025	−4.811	−0.191
	4	−2.329 *	0.756	88.510	0.028	−4.507	−0.151
3rd (Week 9)	0	7.547 *	0.752	88.351	0.000	5.382	9.713
	1	7.534 *	0.755	88.216	0.000	5.359	9.708
	2	2.501 *	0.802	88.747	0.025	0.191	4.811
	4	0.172	0.764	88.074	1.000	−2.028	2.372
4th (Week 12)	0	7.375 *	0.708	88.169	0.000	5.337	9.413
	1	7.362 *	0.711	88.025	0.000	5.314	9.409
	2	2.329 *	0.756	88.510	0.028	0.151	4.507
	3	−0.172	0.764	88.074	1.000	−2.372	2.028

* Level of statistical significance: *p* < 0.05.

**Table 13:** Mann–Whitney U analysis for exercise adherence.

	Adherence Rate
Mann–Whitney U	2720.0000
Wilcoxon W	4205.0000
Z	−2.737
*p*-value	0.006

## Data Availability

Dataset available on request from the authors.

## Abbreviations

The following abbreviations are used in this manuscript:

SPPB	Short Physical Performance Battery
ESE	Exercise Self-Efficacy Scale
TUG	Timed-Up and Go test
ABC	Activities-specific Balance Confidence scale
BREQ-2	Behavioral Regulation in Exercise Questionnaire-2
